# Geographic variation and localised clustering of congenital anomalies in Great Britain

**DOI:** 10.1186/1742-7622-4-14

**Published:** 2007-07-06

**Authors:** Ben G Armstrong, Helen Dolk, Sam Pattenden, Martine Vrijheid, Maria Loane, Judith Rankin, Chris E Dunn, Chris Grundy, Lenore Abramsky, Patricia A Boyd, David Stone, Diana Wellesley

**Affiliations:** 1 Public and Environmental Health Research Unit, London School of Hygiene and Tropical Medicine, Keppel St London WC1E7HT, UK; 2 Faculty of Life and Health Sciences, University of Ulster, Shore Rd Newtownabbey BT370QB, UK; 3 International Agency for Research on Cancer, 150 Cour Albert Thomas, 69372 Lyon Cedex, France; 4 University of Newcastle, School of Population and health Studies, Newcastle upon Tyne NE24HH, UK; 5 Department of Geography, University of Durham, South Road, Durham, DH1 3LE, UK; 6 Congenital Malformations Register, Perinatal Public Health, Northwick Park Hospital, Watford Rd, Harrow HA13UJ, UK; 7 CAROBB, National Perinatal Epidemiology Unit, Institute of Health Sciences, Old Rd, Headington, OX37LF, UK; 8 Paediatric Epidemiology and Community Health (PEACH), Yorkhill Hospital, Glasgow G38SJ, UK; 9 Wessex Clinical Genetics Services, Princess Anne Hospital, Coxford Rd, Southampton S0165YA, UK

## Abstract

**Background:**

Environmental pollution as a cause of congenital anomalies is sometimes suspected because of clustering of anomalies in areas of higher exposure. This highlights questions around spatial heterogeneity (clustering) in congenital anomaly rates. If spatial variation is endemic, then any one specific cluster is less remarkable, though the presence of uncontrolled geographically clustered risk factors is suggested. If rates are relatively homogeneous across space other than around specific hazards, then evidence for these hazards causing the clusters is strengthened. We sought to estimate the extent of spatial heterogeneity in congenital anomaly rates in the United Kingdom.

**Methods:**

The study population covered about one million births from five registers in Britain from 1991–1999. We estimated heterogeneity across four geographical levels: register area, hospital catchment, electoral ward, and enumeration district, using a negative binomial regression model. We also sought clusters using a circular scan statistic.

**Results:**

Congenital anomaly rates clearly varied across register areas and hospital catchments (p < 0.001), but not below this level (p > 0.2). Adjusting for socioeconomic deprivation and maternal age made little difference to the extent of geographical variation for most congenital anomaly subtypes. The two most significant circular clusters (of four ano-rectal atresias and six congenital heart diseases) contained two or more siblings.

**Conclusion:**

The variation in rates between registers and hospital catchment area may have resulted in part from differences in case ascertainment, and this should be taken into account in geographical epidemiological studies of environmental exposures. The absence of evidence for variation below this level should be interpreted cautiously in view of the low power of general heterogeneity tests. Nevertheless, the data suggest that strong localised clusters in congenital anomalies are uncommon, so clusters around specific putative environmental hazards are remarkable when observed. Negative binomial models applied at successive hierarchical levels provide an approach of intermediate complexity to characterising geographical heterogeneity.

## Background

Environmental causes of congenital anomalies may be suspected because of clustering of anomalies in areas of high exposure. For example in the UK two studies have suggested increased rates of congenital anomalies in pregnancies of mothers living close to waste land-fill sites [1,2]. These and similar studies elsewhere [3-10] highlight questions around spatial variation in congenital anomaly rates, also described as heterogeneity or clustering. If spatial variation is endemic, then any one specific cluster is less remarkable, though the presence of uncontrolled geographically clustered risk factors is suggested. If rates are relatively homogeneous across space other than around specific hazards, then evidence for these hazards causing the clusters is strengthened. If there is variation, it is useful to know at what geographical level (local or large-area) it is, and whether it is explained by known risk factors or factors affecting case ascertainment.

The aim of this study was to characterise the extent and level of geographical heterogeneity in congenital anomaly rates in five areas of Britain covered by anomaly registers. In doing this we illustrate application of methods, in particular negative binomial regression, not widely known in epidemiology.

## Analysis

### Data

The collection, cleaning, classification of anomalies by anatomical type, and the source and treatment of data on number of births (denominators) has been described fully elsewhere [1,11,12], so is only briefly summarised here. This analysis concerns cases of selected congenital anomaly among livebirths, fetal deaths from 20 weeks gestation, and terminations of pregnancy following prenatal diagnosis, identified by five regional registers from 1991–1999. We included only anomalies that were well defined and recorded (see footnote to Table 1). We excluded minor anomalies, anomalies which are variably recorded (for example ventricular septal defects) or only recorded by one register (hypospadias), and tumours and neoplasms, metabolic anomalies, and deformations. Mendelian syndromes were excluded[1] as well as syndromes due to maternal exposure to known teratogens such as rubella or valproic acid. Syndromes usually due to new dominant mutations were analysed separately but results are not given here. Other syndromes, mainly those with unknown aetiology or complex or heterogeneous genetic inheritance, are included, but these are not included in either chromosomal or non-chromosomal sub-totals.

**Table 1:** Heterogeneity over geographical areas at each of four hierarchical levels

Area level in hierarchy	Adjustment	Heterogeneity		
		
		CV	90% RR range	p
All non-chromosomal (n = 6959)				
Enumeration district	nothing	0.21	0.68–1.38	0.030
	age+deprivation	0.18	0.72–1.32	0.079
	+register	0.15	0.76–1.26	0.164
	+hosp. catchment	0.08	0.87–1.14	>0.2
Ward	nothing	0.23	0.65–1.41	0.000
	age+deprivation	0.19	0.71–1.33	0.000
	+register	0.13	0.79–1.23	0.006
	+hosp. catchment	0.05	0.92–1.08	>0.2
Hospital catchment	nothing	0.20	0.70–1.35	0.000
	age+deprivation	0.16	0.75–1.28	0.000
	+register	0.10	0.84–1.17	0.000
Register	nothing	0.18	0.72–1.31	0.000
	age+deprivation	0.12	0.81–1.21	0.000

All chromosomal (n = 3152)				
Enumeration district	nothing	0.26	0.61–1.46	0.116
	age+deprivation	0.09	0.86–1.15	>0.2
	+register	0.06	0.90–1.10	>0.2
	+hosp. catchment	0.00	0.99–1.01	>0.2
Ward	nothing	0.26	0.61–1.47	0.000
	age+deprivation	0.17	0.74–1.29	0.029
	+register	0.14	0.79–1.23	0.101
	+hosp. catchment	0.09	0.86–1.15	>0.2
Hospital catchment	nothing	0.13	0.80–1.22	0.000
	age+deprivation	0.09	0.85–1.16	0.003
	+register	0.00	1.00-1.00	>0.2
Register	nothing	0.11	0.82–1.20	0.000
	age+deprivation	0.10	0.83–1.18	0.000

Non chromosomal anomalies: Any cases with the following ICD10 codes excluding cases associated with chromosomal anomalies (for other exclusions see Methods): Q00-03, Q041-042, Q05, Q110-112, Q160, Q172, Q20, Q211-219, Q22-23, Q25-26, Q300-348, Q35, Q36-37, Q390-394, Q41, Q42, Q600-Q605, Q61, Q641-643, Q645, Q71-73, Q77, Q78, Q790-793, Q794

Chromosomal anomalies: ICD10 codes Q90-94, Q96-99

CV: Estimated coefficient of variation of random variation in underlying relative rates (negative binomial model).

90% RR range: estimated range from 5th to 95th percentile of underlying distribution of rates, scaled so that 1 is the average rate.

p: p-value for the significance test for presence of heterogeneity (non-zero variance), according to a likelihood ratio test.

The classification we use for these analyses comprised, in addition to the combined chromosomal and non-chromosomal anomalies, three chromosomal subtypes, 27 non-chromosomal subtypes or groupings of subtypes, and syndromes. We refer to these 31 classifications as "subtypes".

Residential addresses of mothers were classified into areas at four hierarchical levels using their postcode of address at delivery. From the smallest, these were census enumeration district (ED), electoral ward, hospital catchment area, and register. The included registers (from south: Wessex, North West Thames, Oxford, Northern, and Glasgow) cover about 20% of the birth population of the UK. Detailed maps of each register area are provided in a technical report [1]. All registers operated active ascertainment procedures [11]. Geographical areas for the first three registers, which are not fully population-based but cover all hospitals in a geographical area, were identified as electoral wards (areas comprising about 10,000 residents) where at least 80% of births were in hospitals covered by the register. This resulted in an average coverage of 94.6% of births in these three study areas. Births in each ED, stratified by maternal age, were obtained from the Office for National Statistics and General Register Office for Scotland, and adjusted pro-rata in EDs in electoral wards in which less than 100% (i.e. 80–99%) of births were in hospitals covered by the register. There was a total of 10,722 qualifying cases of congenital anomaly and 839,521 births in the study period. (This excludes 122 new dominant mutations from the 10,844 reported previously [1].

To define hospital catchment area, each ward was assigned the code of the hospital where the majority of the births in that ward delivered. Hospital catchment area was preferred a priori to administrative district as a second-level geographical classification because ascertainment completeness was considered likely to be dependent on hospital personnel and procedures. (The two have broadly similar sizes, with average annual births: 1,800 and 1,150 respectively.) The greater ability of hospital catchment area than administrative district to explain variability of anomalies was also confirmed empirically. Hospital catchment was not assigned to cases and births according to hospital of delivery because of the known selective referral of high risk cases between hospitals.

In summary, each anomaly and birth was classified by address of mother at birth in the following geographical hierarchy:

1. Register (n = 5; from 5,000 to 34,000 births annually in each)

2. Hospital catchment area (n = 51; mean 1,800 births annually in each)

3. Electoral ward (n = 1,632; mean 57 births annually in each)

4. Census enumeration district (ED; n = 23,302; mean 4 births annually in each)

Maternal age was classified into the categories <20, 20–34, 35+ for non-chromosomal and <30, 30–34, 35+ for chromosomal. Choice of these group boundaries were informed by the literature on relationships between maternal age and these two classes of anomalies. The Carstairs index for deprivation [13] was calculated for EDs based on 1991 census data on social class of head of household, car ownership, unemployment and overcrowding, standardised to Great Britain. Study population EDs were divided into quintiles according to their Carstairs score.

### Statistical analysis

Methods are summarised here, with technical detail given in the annex.

Cases with missing maternal age (1.3%, 147 cases) and EDs with deprivation index classified as unknown (0.2%, 19 cases) were excluded from all analyses.68 further cases (0.6%) were excluded from the analyses because no births were reported in the ED in the same age group, presumably because of age or residence misclassification.

Variation in congenital anomaly rates across EDs was estimated, allowing for the random (Poisson) variability expected by chance, by assuming a negative binomial regression model of cases and births. The negative binomial regression model, like the Poisson regression model, allows for measured risk factors (here maternal age and area deprivation) and for Poisson variation of anomaly occurrences. However, in addition it allows for random variation of rates across the EDs not explained by either of these things, which would be expected if there were unmeasured risk factors that tended to cluster in EDs. Negative binomial models allow for random variation at only one level of a hierarchy, but by applying it separately at ED, ward, hospital catchment and register level we obtained an estimation of variation at each of these levels.

For easier interpretation we transformed the variation parameter in the negative binomial model to a coefficient of variation (CV: the standard deviation divided by the mean). For example, a CV of 0.1 implies a standard deviation one-tenth of the mean rate. A CV of 0 implies no variation of underlying rates unexplained by measured risk factors or Poisson variation. To further aid interpretation we also transform the estimated underlying CVs to theoretical 5^th ^to 95^th ^percentiles of underlying rates relative to the overall rate [14]. For example a CV of 0.1 corresponds to a 5^th^–95^th ^range in relative rates of 0.84–1.17, i.e. from 84% to 117% of the mean rate. We also report a likelihood ratio test for non-zero CV of underlying rates.

We refer to the CVs as *underlying *CVs of rates across areas, to avoid confusion with the crude CV of rates, which would be much larger, and would mix variation due to Poisson "noise" with actual variation of underlying rates.

The CV and 5^th^–95^th ^percentile range was estimated after adjusting, in turn, for effects of specific risk factors (maternal age and ED deprivation) and for variation across the geographical levels above the one in question. For example, variation across wards is estimated first with no adjustment other than for Poisson variation, then with adjustment for specific risk factors, then also for variation across registers, and finally also for variation across hospital catchment areas. For analysis at ED level, however, to avoid unnecessary technical complexity we adjusted for variation down to hospital catchment rather than ward level. (The complexity would arise from the need to include 1,632 indicator variables for ward or devise a way of working around doing so.)

Spatial clustering not apparent in administrative areas was evaluated using a scan statistic [15]. This method involves drawing circles around each ED centroid, of radius increasing from 0 to a pre-set maximum (in number of births – discussed below), and for each circle comparing the observed with the expected number of cases inside the circle. The most discrepant circle is identified and its statistical significance is assessed by Monte-Carlo simulation allowing for the multiplicity of tests. We calculated expected numbers for each ED adjusting for maternal age and ED deprivation (known risk factors), and hospital catchment area (suspected source of ascertainment differences). Because we adjusted for hospital catchment area, we sought clusters only below this level, and thus set the maximum radius of the scan circles to the average size (defined in terms of births) of a catchment area.

Results statistically significant at p < 0.05 are referred to as "statistically significant", but we do not mean to imply by this that p < 0.05 signifies a specially important cut-point of significance.

## Results

Estimates of underlying heterogeneity at each of the four hierarchical levels are shown in Table 1 for combined non-chromosomal and combined chromosomal anomalies. For chromosomal anomalies variation between registers is present and significant, even after adjusting for age and deprivation. However, adjustment for maternal age and area deprivation reduces heterogeneity between EDs, wards, and hospital catchments, and at no level is the heterogeneity significant after allowing for variation between registers (Table 1). For Down Syndrome specifically (data not shown), regional variation (CV = 0.10) was removed entirely after adjustment for maternal age.

Unadjusted non-chromosomal anomaly rates show significant heterogeneity at all levels. The variation is little reduced on adjusting for effects of maternal age and area deprivation, but variation across wards and EDs is substantially reduced on adjusting for variation across hospital catchment areas. In other words, after adjustment there is evidence for clustering at the region and hospital catchment area level, but not below it.

We also estimated heterogeneity for 31 specific anomaly subtypes or groupings of them [1], but for brevity show only fully adjusted estimates for the 11 judged most important a priori (Table 2). Two subtypes showed nominally significant (p < 0.05) heterogeneity over EDs after full adjustment: neural tube defects and specifically spina bifida. Variation over EDs of gastroschisis was close to significant (p = 0.051). There was no subtype with significant variation over wards. There was significant variation over hospital catchment areas (allowing for variation over registers) in three specific anomaly subtypes (hydrocephaly, congenital heart disease, cleft lip), and over registers in ten subtypes [1].

**Table 2:** Summary of geographic heterogeneity for specific anomaly groups

Anomaly	Level	N cases	Heterogeneity		
			
			CV	90% RR range	P
Neural tube	ED	1163	0.60	0.25–2.15	0.009
defects	ward		0.00	1.00-1.00	>0.2
	hosp		0.06	0.90–1.11	>0.2
	register		0.05	0.91–1.09	0.143
Hydrocephaly	ED	373	0.54	0.30–2.03	>0.2
	ward		0.00	1.00-1.00	>0.2
	hosp		0.33	0.52–1.60	0.001
	register		0.08	0.87–1.14	>0.2
Congenital	ED	2211	0.01	0.99–1.01	>0.2
heart disease	ward		0.00	1.00-1.00	>0.2
	hosp		0.12	0.81–1.21	0.006
	register		0.26	0.61–1.46	0.000
Cleft lip	ED	662	0.57	0.28–2.08	0.091
	ward		0.28	0.59–1.49	0.115
	hosp		0.27	0.60–1.48	0.000
	register		0.09	0.85–1.16	0.045
Cleft palate	ED	359	0.00	1.00-1.00	>0.2
	ward		0.00	1.00-1.00	>0.2
	hosp		0.00	1.00-1.00	>0.2
	register		0.22	0.67–1.39	0.016
Bilateral	ED	119	1.28	0.01–3.58	0.123
renal	ward		0.73	0.16–2.43	0.077
agenesis	hosp		0.00	1.00-1.00	>0.2
	register		0.36	0.49–1.66	0.013
Limb reduction	ED	348	0.68	0.19–2.32	0.174
	ward		0.00	1.00-1.00	>0.2
	hosp		0.07	0.88–1.13	>0.2
	register		0.15	0.76–1.27	0.097
Diaphragmatic	ED	236	0.91	0.08–2.80	0.142
hernia	ward		0.00	1.00-1.00	>0.2
	hosp		0.00	1.00-1.00	>0.2
	register		0.13	0.79–1.23	0.176
Gastroschisis	ED	244	1.07	0.04–3.15	0.051
	ward		0.00	1.00-1.00	>0.2
	hosp		0.00	1.00-1.00	>0.2
	register		0.13	0.79–1.23	0.079
Multiple	ED	820	0.32	0.53–1.59	>0.2
anomalies	ward		0.00	1.00-1.00	>0.2
	hosp		0.09	0.86–1.15	>0.2
	register		0.30	0.56–1.54	0.000
Syndromes	ED	377	1.27	0.01–3.55	0.001
	ward		0.31	0.55–1.56	>0.2
	hosp		0.00	1.00-1.00	>0.2
	register		0.26	0.62–1.46	0.000

Specific congenital anomaly subtypes analysed but not shown in this table:

A: selected a priori but without evidence for heterogeneity at any level (p > 0.2): all digestive system atresias, cystic kidney disease, Omphalocele, Down syndrome (n = 4)

B: not selected a priori: anencephaly, spina bifida, anomalies of cardiac chambers, transposition of great vessels, malformations of cardiac septa, atrioventricular septal defect, tetralogy of fallot, malformations of valves, hypoplastic left heart, malformations of great arteries and veins, coarctation of aorta, tracheo-oesophageal fistula and oesophageal atresia, small intestine atresia, anorectal atresia, Patau's syndrome, Edwards' syndrome (n = 16)

As with non-chromosomal anomalies combined (Table 1), little of the variation in subtypes across register areas was explained by maternal age or socioeconomic deprivation differences (details not shown). The exceptions were combined digestive system atresias, for which all regional variation was removed after adjustment, gastrochisis (from CV = 0.29 to 0.13), and neural tube defects (from 0.11 to 0.05).

The group of syndromes with unknown etiology (as described in Data above) showed nominally significant (p < 0.05) heterogeneity over EDs after adjusting for effects of maternal age, area deprivation, and variation over registers and hospital catchment areas. This high variation was driven by more than expected EDs with two or three cases, of which half were pairs of cases to the same mother.

Applying the circular scan statistic to the overall data for non-chromosomal and chromosomal anomalies and the 31 specific subtypes (including syndromes) yielded two clusters achieving nominal statistical significance at p < 0.05 (congenital heart disease, n = 6, p = 0.04; ano-rectal atresia and stenosis, n = 4, p = 0.003). The cluster of ano-rectal anomalies was in a 450 m circle including 18 EDs, with four observed cases and 0.03 expected. On further examination, it was found that three of these were to the same mother (siblings). The cluster of congenital heart disease anomalies was in a 230 m circle including seven EDs, with six observed cases and 0.26 expected. These six cases included two to the same mother. The scan statistic did not identify any circular cluster of non-chromosomal syndromes as being unusual.

We compared heterogeneity of rates across hospital catchments with that across administrative districts. There was highly significant heterogeneity of all non-chromosomal anomalies across districts and across hospital catchment areas. However, hospital catchment explained more deviance per degree of freedom, and remained significant after allowing for district effects (p = 0.02), whereas district was not significant after allowing for hospital catchment effects (p = 0.16).

## Discussion

There was clear heterogeneity in non-chromosomal congenital anomaly rates between register regions as shown in more detail previously [11]. Only a small part of this was explained by differences in maternal age and deprivation. Some of the remaining regional variation may reflect real differences in other risk factors, for example nutritional factors for spina bifida, but some variation is likely to be due to ascertainment differences [11]. For chromosomal anomalies as a whole, we found some evidence of regional variation, but not for Down Syndrome specifically after maternal age had been taken into account. Regional and hospital variation in other chromosomal anomalies may be due to differences in prenatal screening methods, which can pick up anomalies that would not otherwise be evident until later life. However, there was no regional variation in the well diagnosed Edwards and Patau syndrome specifically.

This paper shows clear evidence for heterogeneity between hospital catchment areas also for non-chromosomal anomalies in general (90% range of underlying relative risks 0.84–1.17) and some subtypes (hydrocephaly, congenital heart disease, cleft lip). These may well have been caused at least in part by differences in ascertainment. If this is true, it would suggest that adjustment for hospital catchment area may be necessary to control for ascertainment bias in studies of congenital anomalies in relation to putative risk factors. It is likely that some ascertainment differences between hospitals would be present in most registers. This was shown also in a geographical analysis in New York State[16] That study however looked at individual birth hospital of delivery, which could also reflect selection of high risk pregnancies by some hospitals, whereas our method of comparing catchment areas distinguished ascertainment (and other area) differences from such selection effects. The types of differences between hospitals reporting to the same register can include hospital practices with regard to prenatal and neonatal screening, the thoroughness with which newborns are examined for more minor anomalies and how these are recorded, referral practices, linkage with specialist services such as paediatric cardiology, how efficiently and accurately congenital anomaly diagnoses can be retrieved from hospital databases, and the quality of collaboration between the hospital and the register.

Below hospital catchment area level, there was very little evidence for heterogeneity or clustering. The finding that syndromes and two non-chromosomal subtypes (neural tube defects and specifically spina bifida) had nominally significant heterogeneity across EDs is compatible with chance findings from multiple testing. Further surveillance should establish whether this finding holds. The borderline significant gastroschisis finding is of interest given the increasing trends in prevalence over the last few decades reported elsewhere and rather strong regional differences in prevalence [17-22].

Our results should be interpreted in the light of the limitations in power to detect clusters. In particular for rarer congenital anomalies, geographical heterogeneity (clustering) would have to have been substantial for our tests to be able to detect it. For example, we estimated a 5^th^–95^th ^percentile range of underlying limb reduction (n = 348) rates across EDs, relative to the overall rate, of 0.19–2.32 (a 12-fold range), but the significance level was only p = 0.17. Nevertheless, there need not be many EDs with more than one case to cause large and statistically significant heterogeneity. For example, exploratory analyses showed that for heterogeneity to be very strongly significant (p = 0.002) for a rare anomaly (121 cases), it was sufficient to have just two EDs with two anomalies and one with three anomalies. Indeed, our strongest finding of variation related to syndromes, where some EDs had more than one case from the same family. Much smaller variation is detectable between registers, and somewhat so between hospital catchment areas. For example the 5^th^–95^th ^RR range estimated between registers for limb reduction was just 0.76–1.27, but this approached significance at p = 0.097.

The scan statistic complemented the other tests of geographic variation by picking up clustering at geographical levels other than ED and ward, and by not respecting ward boundaries in examining areas larger than ED size. The two nominally significant "clusters" found by the scan statistic should be interpreted cautiously in view of the large number of congenital anomaly types tested, and the dominance of the more unusual cluster by one family. That three of the four cases are to the same mother does not preclude a common environmental explanation (in particular an exposure specific to this home), but it could also be familial susceptibility to the environmental factor, or alternatively familial inheritance unrelated to any local environmental exposure. Overall therefore we found a remarkable sensitivity of all clustering methods to family clusters, which are particularly difficult to interpret in relation to possible environmental factors. We performed careful cleaning of the data for duplicate registrations, but it can be easily seen from our consideration of familial cases that duplicate registrations would cause detectable clustering, an important warning for further research.

The absence of evidence for general heterogeneity below the hospital catchment area cannot be taken as evidence against a modest degree of more specific heterogeneity, for example between areas of different deprivation level [1], or specific clustering close to sources of environmental exposures. Data from these registers contributed towards the finding of clustering around hazardous waste land-fill sites [23], and reanalysis with these data have confirmed this for non-chromosomal anomalies although with reduced odds ratios [1]. Further, the absence of evidence for generalised local clustering reassures that the excess found close to waste landfill sites is indeed as unusual as conventional statistical methods suggest.

We used statistical methods of intermediate complexity. Could more sophisticated spatial analysis reveal more of interest? It had been our intention to explore spatial patterns further using Bayesian and other "multi-level" hierarchical models, including spatial adjacency models (investigating whether adjacent areas have similar rates). However, exploratory analyses using even the simpler of such models to investigate spatial patterning in variation below hospital catchment area (random effects between wards or EDs) suggested that estimates of heterogeneity were often sensitive to specific model specification, in particular "prior" distribution for variance. This seems likely to be a further consequence of the sparseness of occurrence of anomalies in EDs and wards – it is too much to expect these data to give information on subtle spatial patterns in rates at the local level. The problem seems likely to be less for analyses of all anomalies combined than for specific anomaly groups, and less for variation between wards than variation between EDs, but we sought an approach applicable routinely in all analyses. We did not pursue use of these models at higher geographic levels (investigate whether adjacent hospital catchments have similar rates), because the likelihood of variation between hospital catchments being artefacts of case ascertainment made interpretation less interesting.

We sought here to describe geographical variation. Another use of models incorporating geographical variation is to allow for it when addressing specific risk factors in "ecologic correlation studies". In general ignoring such variation, if present, exaggerates certainty in such analyses, and in some situations can bias the estimated effects. For this use the negative binomial model – which can only include random variation at one level of the hierarchy in any one analysis and does not incorporate other modes of spatial autocorrelation (e.g. similarity in adjacent areas) – is more limited. Models which do incorporate spatial structure more fully reduce the risk of underestimating uncertainty, though where data is sparse conclusions may depend strongly on prior information rather the empirical observation of spatial structure.

Our results give encouragement that well-cleaned register data, limited to a predefined set of well diagnosed and reported congenital anomalies, can be adequate to investigate putative specific environmental causes of anomalies for which exposure varies geographically below the hospital catchment area level. Major data errors that would bedevil such studies would have been likely to have shown up in this study as localised clustering. Such studies would generally need estimates of exposure to fine geographical resolution and should, where possible, control for "hospital catchment" effects.

## Conclusion

We conclude with two substantive and two methodological points. First, there is clear evidence for variation (clustering) at hospital catchment area and register level, particularly for non-chromosomal anomalies. This variation, at least in part, is likely to be due to ascertainment differences. Secondly, There is little evidence for more localised generalised clustering, and the few suggestive results were explained by anomalies in the same family – though power was limited, especially for rarer congenital anomalies.

From a methodological point of view, we propose that geographical studies of anomalies should control for variation by hospital catchment area. Finally, negative binomial models applied at successive hierarchical levels provide an approach of intermediate complexity to characterising geographical heterogeneity which is fairly straightforward to apply, is robust to low event rates, and can yield epidemiologically interpretable summaries of heterogeneity.

## Appendix

Details of statistical analysis

The data comprised, for each maternal age group i in each enumeration district (ED) j:

• the number of anomalies of each type y_ij_

• the number of births n_ij_

And for each ED:

• A measure of area socio-economic deprivation derived from census variables – Carstairs quintile (1–5) c_j_

• The electoral ward w_j _= 1...1632 within which the ED lies

• The hospital catchment h_j _= 1...51 within which the ED lies

• The register region r_j _= 1...5 within which the ED lies

The analysis followed the following steps:

Preliminary: calculating expected number of cases at ED level adjusting for maternal age group and area deprivation:

• assume y_ij_~negbin(n_ij_μ_ij_, τ^2^), log⁡(μij)=αi+βcj
 MathType@MTEF@5@5@+=feaafiart1ev1aaatCvAUfKttLearuWrP9MDH5MBPbIqV92AaeXatLxBI9gBaebbnrfifHhDYfgasaacH8akY=wiFfYdH8Gipec8Eeeu0xXdbba9frFj0=OqFfea0dXdd9vqai=hGuQ8kuc9pgc9s8qqaq=dirpe0xb9q8qiLsFr0=vr0=vr0dc8meaabaqaciaacaGaaeqabaqabeGadaaakeaacyGGSbaBcqGGVbWBcqGGNbWzcqGGOaakiiGacqWF8oqBdaWgaaWcbaGaemyAaKMaemOAaOgabeaakiabcMcaPiabg2da9iab=f7aHnaaBaaaleaacqWGPbqAaeqaaOGaey4kaSIae8NSdi2aaSbaaSqaaiabdogaJnaaBaaameaacqWGQbGAaeqaaaWcbeaaaaa@40E6@

• estimate α^i
 MathType@MTEF@5@5@+=feaafiart1ev1aaatCvAUfKttLearuWrP9MDH5MBPbIqV92AaeXatLxBI9gBaebbnrfifHhDYfgasaacH8akY=wiFfYdH8Gipec8Eeeu0xXdbba9frFj0=OqFfea0dXdd9vqai=hGuQ8kuc9pgc9s8qqaq=dirpe0xb9q8qiLsFr0=vr0=vr0dc8meaabaqaciaacaGaaeqabaqabeGadaaakeaaiiGacuWFXoqygaqcamaaBaaaleaacqWGPbqAaeqaaaaa@2FE9@, β^
 MathType@MTEF@5@5@+=feaafiart1ev1aaatCvAUfKttLearuWrP9MDH5MBPbIqV92AaeXatLxBI9gBaebbnrfifHhDYfgasaacH8akY=wiFfYdH8Gipec8Eeeu0xXdbba9frFj0=OqFfea0dXdd9vqai=hGuQ8kuc9pgc9s8qqaq=dirpe0xb9q8qiLsFr0=vr0=vr0dc8meaabaqaciaacaGaaeqabaqabeGadaaakeaaiiqacuWFYoGygaqcaaaa@2E63@ by maximum likelihood, and hence expected number of cases ei=∑inijμ^ij=∑inijexp⁡(α^i+β^cj)
 MathType@MTEF@5@5@+=feaafiart1ev1aaatCvAUfKttLearuWrP9MDH5MBPbIqV92AaeXatLxBI9gBaebbnrfifHhDYfgasaacH8akY=wiFfYdH8Gipec8Eeeu0xXdbba9frFj0=OqFfea0dXdd9vqai=hGuQ8kuc9pgc9s8qqaq=dirpe0xb9q8qiLsFr0=vr0=vr0dc8meaabaqaciaacaGaaeqabaqabeGadaaakeaacqWGLbqzdaWgaaWcbaGaemyAaKgabeaakiabg2da9maaqafabaGaemOBa42aaSbaaSqaaiabdMgaPjabdQgaQbqabaacciGccuWF8oqBgaqcamaaBaaaleaacqWGPbqAcqWGQbGAaeqaaaqaaiabdMgaPbqab0GaeyyeIuoakiabg2da9maaqafabaGaemOBa42aaSbaaSqaaiabdMgaPjabdQgaQbqabaGccyGGLbqzcqGG4baEcqGGWbaCcqGGOaakcuWFXoqygaqcamaaBaaaleaacqWGPbqAaeqaaOGaey4kaSIaf8NSdiMbaKaadaWgaaWcbaGaem4yam2aaSbaaWqaaiabdQgaQbqabaaaleqaaOGaeiykaKcaleaacqWGPbqAaeqaniabggHiLdaaaa@54B7@

Step 1: estimation of heterogeneity of underlying rates over EDs

Step 1a: crude rates

• compute total number of cases y_j _and births n_j _in each ED.

• fit y_j_~negbin(n_j_μ, τ^2^), estimate τ by maximum likelihood

• carry out likelihood ratio test against τ = 0

Step 1b: adjusting for maternal age and deprivation

• Repeat step 1 replacing births n_j _with expected cases e_j_

Step 1c: adjusting for maternal age, deprivation and register

• Repeat step 1a but include a register indicator in the linear predictor; ie replace μ by μj=exp⁡(α+γrj)
 MathType@MTEF@5@5@+=feaafiart1ev1aaatCvAUfKttLearuWrP9MDH5MBPbIqV92AaeXatLxBI9gBaebbnrfifHhDYfgasaacH8akY=wiFfYdH8Gipec8Eeeu0xXdbba9frFj0=OqFfea0dXdd9vqai=hGuQ8kuc9pgc9s8qqaq=dirpe0xb9q8qiLsFr0=vr0=vr0dc8meaabaqaciaacaGaaeqabaqabeGadaaakeaaiiGacqWF8oqBdaWgaaWcbaGaemOAaOgabeaakiabg2da9iGbcwgaLjabcIha4jabcchaWjabcIcaOiab=f7aHjabgUcaRiab=n7aNnaaBaaaleaacqWGYbGCdaWgaaadbaGaemOAaOgabeaaaSqabaGccqGGPaqkaaa@3E3E@

Step 1d: adjusting for maternal age, deprivation and hospital catchment

• Repeat step 1a but include a hospital catchment indicator in the linear predictor; ie replace μ by μj=exp⁡(α+δhj)
 MathType@MTEF@5@5@+=feaafiart1ev1aaatCvAUfKttLearuWrP9MDH5MBPbIqV92AaeXatLxBI9gBaebbnrfifHhDYfgasaacH8akY=wiFfYdH8Gipec8Eeeu0xXdbba9frFj0=OqFfea0dXdd9vqai=hGuQ8kuc9pgc9s8qqaq=dirpe0xb9q8qiLsFr0=vr0=vr0dc8meaabaqaciaacaGaaeqabaqabeGadaaakeaaiiGacqWF8oqBdaWgaaWcbaGaemOAaOgabeaakiabg2da9iGbcwgaLjabcIha4jabcchaWjabcIcaOiab=f7aHjabgUcaRiab=r7aKnaaBaaaleaacqWGObaAdaWgaaadbaGaemOAaOgabeaaaSqabaGccqGGPaqkaaa@3E28@

Step 2: estimation of heterogeneity of underlying rates over wards

As for step 1 bt replacing EDs by wards, hence

• compute total number of cases y_k_, births n_k _and expected cases e_k _in each ward k = 1...1632

• etc

Steps 3 and 4: estimation of heterogeneity of underlying rates over hospital catchments and register areas

As for step 1 replacing ED by hospital catchment and register area.

Interpretation of τ as CV of underlying rate.

For example, over EDs we assume y_j_~negbin(n_j_μ, τ^2^). This implies mean = n_j_μ_j_, variance = n_j_μ_j_+(n_j_μ_j_)^2^τ^2^. The first term of the expression for variance is that expected with Poisson variation (ie no variation of underlying rates); the second term is the additional variation when there is variation over EDs in underlying rates. Since mean = n_j_μ_j_, for this second term CV = (n_j_μ_j_)τ/n_j_μ_j _= τ.

## Abbreviations

ED Enumeration District

CV Coefficient of Variation

## Competing interests

The author(s) declare that they have no competing interests.

## Authors' contributions

BA designed and led statistical analysis and drafted most of manuscript. HD designed and led overall study and drafted parts of manuscript. SP carried out some statistical analyses and contributed to statistical design. MV designed and managed overall study and assembled and cleaned data. ML carried out some "scan" analyses. JR oversaw data quality, provided Northern date and compiled combined data. CD and CG carried out geographic information system linkages. LA, PB, DS, and DW oversaw data quality and provided data from NW Thames, Oxford, Glasgow, and Wessex, respectively. All authors read and approved the final manuscript.
